# Risk factors and their association network for young adults’ suicidality: a cross-sectional study

**DOI:** 10.1186/s12889-024-18860-9

**Published:** 2024-05-22

**Authors:** Junsong Lu, Yan Jin, Sugai Liang, Qiang Wang, Xiaojing Li, Tao Li

**Affiliations:** 1 Affiliated Mental Health Centre & Hangzhou Seventh People’s Hospital, Zhejiang University School of Medicine, Hangzhou, Zhejiang 310013 China; 2 School of Humanities and Social Science, The Chinese University of Hong Kong, Shenzhen, Guangdong 518712 China; 3 Liangzhu Laboratory, MOE Frontier Science Center for Brain Science and Brain-machine Integration, State Key Laboratory of Brain-machine Intelligence, Zhejiang University, 1369 West Wenyi Road, Hangzhou, 311121 China; 4 NHC and CAMS Key Laboratory of Medical Neurobiology, Zhejiang University, Hangzhou, 310058 China; 5 Mental Health Centre & Psychiatric Laboratory, State Key Laboratory of Biotherapy, West China Hospital, Sichuan University, Chengdu, Sichuan 610041 China

**Keywords:** Suicide, Machine learning, Network analysis, Risk factor, Prediction

## Abstract

**Background:**

Understanding the intricate influences of risk factors contributing to suicide among young individuals remains a challenge. The current study employed interpretable machine learning and network analysis to unravel critical suicide-associated factors in Chinese university students.

**Methods:**

A total of 68,071 students were recruited between Sep 2016 and Sep 2020 in China. Students reported their lifetime experiences with suicidal thoughts and behaviors, categorized as suicide ideation (SI), suicide plan (SP), and suicide attempt (SA). We assessed 36 suicide-associated factors including psychopathology, family environment, life events, and stigma. Local interpretations were provided using Shapley additive explanation (SHAP) interaction values, while a mixed graphical model facilitated a global understanding of their interplay.

**Results:**

Local explanations based on SHAP interaction values suggested that psychoticism and depression severity emerged as pivotal factors for SI, while paranoid ideation strongly correlated with SP and SA. In addition, childhood neglect significantly predicted SA. Regarding the mixed graphical model, a hierarchical structure emerged, suggesting that family factors preceded proximal psychopathological factors, with abuse and neglect retaining unique effects. Centrality indices derived from the network highlighted the importance of subjective socioeconomic status and education in connecting various risk factors.

**Conclusions:**

The proximity of psychopathological factors to suicidality underscores their significance. The global structures of the network suggested that co-occurring factors influence suicidal behavior in a hierarchical manner. Therefore, prospective prevention strategies should take into account the hierarchical structure and unique trajectories of factors.

**Supplementary Information:**

The online version contains supplementary material available at 10.1186/s12889-024-18860-9.

## Background

Suicide represents a major global public health issue [1], with China experiencing particularly high rates [2], especially among young people aged 15–35 years [3]. This demographic category is pivotal in various research studies, encompassing young individuals navigating significant life stages. They face critical decisions regarding education and career choices, alongside psychological and social developments as they transition from adolescence to adulthood. They may seek financial independence while grappling with student loans and employment instability. Mental health also becomes particularly crucial during this phase. Therefore, research and policy development targeting this age group are essential for facilitating a smooth transition into adulthood and enhancing overall well-being [4]. In China, over the past 20 years, the prevalence rates of SI, SP, and SA among young people are 10·7-32%, 9-11%, and 2·7%-5% respectively [3, 5]. Despite efforts to address this issue, there has been a plateau in the decline of suicide rates in China [6], presenting ongoing challenges for suicide prevention. Notably, urban and rural suicide mortality rates among young people ranging from 1·56%-2·52% per 100,000 population [1, 3]. Furthermore, young people in China have shown the fastest increase in suicide risk [7]. Suicidality, encompassing suicidal ideation (SI), suicide plan (SP), and suicide attempt (SA), is one of the strongest predictors of future psychiatric morbidity and mortality [8, 9], leading to enduring vulnerability to physical and mental health issues [10]. Such outcomes contribute to heightened social burdens and legal complexities. Thus, timely identification of risk factors for suicidality is crucial for effective intervention and a comprehensive understanding of suicide-related phenomena.

Previous studies have identified numerous risk factors associated with suicidality among young people, spanning social, family, individual, and psychiatric factors [11–13]. These factors are often organized within stress-diathesis models [11], which emphasize both proximal and distal effects of multiple factors [14]. Researchers have attempted to build effective suicide risk prediction models to detect risk factors for early detection and prevention [8]. However, traditional statistical methods have encountered limited success due to the intricate interrelationships among risk factors [15, 16]. For example, the area under the receiver operating characteristic curve (AUC) for predicting suicide risk ranged from 0·56 − 0·58 over the past 50 years [16], underscoring the importance of modeling interactive relationships among diverse risk factors and taking into account the nuanced context within which each risk factor exerts its influence. For example, mental disorders strongly predict SP and SA, but their predictive power is largely attenuated after controlling for SI [16]. Furthermore, it’s important to note that no single risk factor, even those with substantial relative effects such as exhibiting high odds ratios in logistic regression, can accurately predict suicidal behavior when considered in isolation [17]. Considering intricate interactions between factors and the constraints of traditional models, a more promising approach would shift the focus towards multifactorial data and algorithms capable of modeling nonlinear dynamics of risk factors. While machine learning has been utilized in suicide research, studies often prioritize prediction over explanation due to the low interpretability of black box models [18]. Additionally, few explore the global structures that reveals relationships between factors or the relationship between different facets of suicidal behavior. Our study aims to fill these gaps by providing local explanations using interpretable machine learning and leveraging graphical models to construct an associative network, examining how these factors cluster and contribute to suicidal behavior.

The integration of the Online Health Survey (OHS) system with machine learning techniques presents a unique opportunity to identify and evaluate suicide risk factors in a large, multi-domain dataset of young people on Chinese campuses. We hypothesized that psychopathologies and specific adverse life experiences stemming from family environments and social connections serve as important predictors of suicidality in young people. In addition, these factors are broadly associated with each other in a nonlinear pattern that traditional models struggle to capture effectively. Embracing these methodological advancements and multifactorial data holds the potential to revolutionize our understanding of suicide risk factors and pave the way for more effective interventions and support systems in mental health care.

## Methods

### Data source and participants

The current study is retrospective, with all data sourced from the OHS database, managed by West China Hospital, Sichuan University. Following ethical approval from the Institutional Review Board (i.e., the Ethics Committee of West China Hospital, Sichuan University), the study was authorized to use relevant, de-identified data devoid of any private information, strictly for academic purposes. This included ensuring consistency in age criteria (15–35 years) during the data extraction process. The OHS system used self-reported questionnaires and scales to measure factors related to the physical and mental health of young people. The survey was conducted online and included participants who were freshmen enrolled at a comprehensive university in China from 2016 to 2020. The sample consisted of 68,071 participants with an average age of 20 years. The survey included 36 factors associated with suicidality. These factors were selected from validated and published measurement tools by a team of professional clinicians, psychiatrists, and psychologists. For more detailed descriptions of the OHS study, refer to the Appendix p.3–7.

### Procedures

The suicidality of each participant was evaluated using the suicidal ideation and behaviors module from the Chinese version of the World Mental Health Composite International Diagnostic Interview (CIDI). The module includes questions on the presence of suicidal ideation, plans, and attempts during one’s lifetime.

The 36 suicide-associated factors evaluated in this study include sociodemographic factors, family factors (e.g. family abuse and neglect), life events (e.g. life stress and traumatic events), altitude towards mental illness (e.g., stigma and help-seeking behavior), basic physical health conditions (e.g. chronic diseases and disability), and psychopathology factors (e.g. depression, anxiety, and psychoticism). The validated questionnaires and scales used to evaluate these factors include CIDI for ideation and behaviors, Symptoms Checklist-90-Revised (SCL-90-R) for psychiatric symptoms, the Patient Health Questionnaire-15 (PHQ-15) for somatic symptoms, the Patient Health Questionnaire-9 (PHQ-9) for depression symptoms, and the adolescent self-rating life events checklist (ASLEC) for life events. More detailed information about each scale and variable can be found in the Appendix (p.3–7).

### Model training and validation

All analyses were conducted by using R version 4.2.2, incorporating packages such as tidyverse, MatchIt, xgboost, Matrix, caret, optmatch, SHAPforxgboost, pROC, tictoc, and unbalanced. Given the prevalence rates of suicide ideation, plans, and attempts differed substantially from the 50% base rate, the sample turned out to be an imbalanced dataset. The imbalanced dataset problem can lead to biased model predictions, with the model being more likely to predict the majority class. To address this, we used random undersampling to create balanced datasets for each suicide variable, split into a training set (80%) and a validation set (20%) for machine learning models. This ensures accurate prediction of the minority class and avoids bias towards the majority class.

In this study, we chose XGBoost as a classifier to predict suicidal behavior and to identify important suicide-associated factors. XGBoost implements the gradient boosting ensemble algorithm [19], and is advantageous for exploiting non-linear and complex relationships between features and the response. Optimal hyperparameters of XGBoost were selected using a grid search on the training set via five-fold cross-validation repeated five times. The final model was chosen according to the one standard error rule and evaluated using AUC. A model with good discrimination is necessary before explaining its predictions.

### Local explanations and global understanding of suicide-associated factors

We sought to gain a more comprehensive understanding of the relationship between suicide-associated factors and suicidality. To achieve this, we analyzed the effect of individual factors and explored the interplay between various factors to form a more holistic picture. By doing so, we were able to provide a more nuanced understanding of the impact of different factors on suicidality. A more detailed explanation of this part could be found in the Appendix (p.7).

Local explanations of individual factors were computed based on Shapley additive explanation (SHAP) values [20], which were developed to interpret black-box models like XGBoost. Mean absolute SHAP values for each feature in each model were calculated to compare the relative importance of various factors in predicting suicide ideation, plan, and attempt. Then, the individual SHAP values of each feature were plotted in a SHAP summary plot for each observation to show the distribution of feature contributions to the model out. The summary plot provided a clear relationship between these factors and suicidal behavior.

To understand the interplay among suicide-related factors, a mixed graphical model (MGM) was used in addition to local explanations [21]. MGM is a regularized network estimation method and estimate parsimonious and interpretable relationships between variables [22]. Each node in the MGM represents a factor, and each edge between two nodes represents a non-zero association. To reduce spurious associations, the MGM uses graphical LASSO regularization and EBIC model selection. The top 15 factors with the highest mean absolute SHAP values for each suicidal behavior were used to estimate the MGM. Three centrality indices (strength centrality, closeness centrality, and betweenness centrality) were calculated for each node to measure its importance in the network.

## Results

The total sample consisted of 68,071 cases with a mean age of 20 5 years (SD 3∙5), including 35,282 [52%] females and 32,789 [48%] males. Demographic data are summarized in Table 1. Overall, 8,732 (12·8% [95% CI 12·6–13·1]) participants reported lifetime suicide ideation (SI), 2,217 (3·3% [3·1–3·4]) participants reported lifetime suicide plan (SP), and 810 (1·2% [1·1–1·3]) participants reported lifetime suicide attempt (SA). Along the process from ideation to attempt, the number of people engaged in suicidality sharply decreases. The morbidity of suicidality is generally higher in women (57·5% in SI, 62·1% in SP, 62·3% in SA) than in men. Participants with higher levels of education have lower incidence rates of suicidality. In term of family factors, there is little difference among the frequencies of the four parents’ education level groups in SA, but the frequencies of SI/SP/SA are the lowest in the low-educated father group and the highest in the higher-educated father group. Moreover, the morbidity rates of all groups of SI/SP/SA without a left-behind experience are generally higher than the groups with left-behind experience. Additionally, the prevalence of mental health disorders is significantly associated with suicidality.

**Table 1:** Demographic data of the total sample and the three balanced samples

	Total sample (*n*=68,071)		Suicide ideation (*n*=17,464)		Suicide plan (*n*=4434)		Suicide attempt (*n*=1620)	
	Cases	Controls	Cases	Controls	Cases	Controls	Cases	Controls
**Participants**	9111 (13·38%)	58,960 (86·62%)	8732 (50·00%)	8732 (50·00%)	2217 (50·00%)	2217 (50·00%)	810 (50·00%)	810 (50·00%)
Female*	5223 (7·67%)	30,059 (44·16%)	5019* (28·74%)	4448 (25·47%)	1377* (31·06%)	1129 (25·46%)	505 (31·17%)	431 (26·60%)
**Age, years**	3888 (5·71%)	28,901 (42·46%)	3713 (21·26%)	4284 (24·53%)	840 (18·94%)	1088 (24·54%)	305 (18·83%)	379 (23·40%)
**Cohort**								
2016*	1742 (2·56%)	9297 (13·66%)	1634* (9·36%)	1397 (8·00%)	441 (9·95%)	365 (8·23%)	155 (9·57%)	135 (8·33%)
2017*	1850 (2·72%)	10,431 (15·32%)	1797* (10·29%)	1528 (8·75%)	435 (9·81%)	395 (8·91%)	155 (9·57%)	140 (8·64%)
2018	2139 (3·14%)	13,197 (19·39%)	2071 (11·86%)	1919 (10·99%)	502 (11·32%)	472 (10·65%)	171 (10·56%)	196 (12·10%)
2019	1863 (2·74%)	12,285 (18·05%)	1777 (10·18%)	1832 (10·49%)	484 (10·92%)	483 (10·89%)	185 (11·42%)	162 (10·00%)
2020*	1517 (2·23%)	13,750 (20·20%)	1453* (8·32%)	2056 (11·77%)	355 (8·01%)	502 (11·32%)	144 (8·89%)	177 (10·93%)
**Education level**								
Undergraduate	7268 (10·68%)	33,091 (48·61%)	6983* (39·99%)	4905 (28·09%)	1886* (42·53%)	1341 (30·24%)	657* (40·56%)	493 (30·43%)
Graduate	1580 (2·32%)	21,237 (31·20%)	1506* (8·62%)	3156 (18·07%)	280* (6·31%)	717 (16·17%)	132* (8·15%)	268 (16·54%)
Doctorate*	263 (0·39%)	4632 (6·80%)	243* (1·39%)	671 (3·84%)	51* (1·15%)	159 (3·59%)	21 (1·30%)	49 (3·02%)
**Father’s education level**								
Junior high school and below	3647 (5·36%)	24,809 (36·45%)	3455 (19·78%)	3674 (21·04%)	848 (19·12%)	949 (21·40%)	359 (22·16%)	351 (21·67%)
Senior high school and above	5464 (8·03%)	34,151 (50·17%)	5277 (30·22%)	5058 (28·96%)	1369 (30·88%)	1268 (28·60%)	451 (27·84%)	459 (28·33%)
**Mother’s education level**								
Junior high school and below*	4238 (6·23%)	29,315 (43·07%)	4026* (23·05%)	4384 (25·10%)	978 (22·06%)	1116 (25·17%)	407 (25·12%)	406 (25·06%)
Senior high school and above*	4873 (7·16%)	29,645 (43·55%)	4706* (26·95%)	4348 (24·90%)	1239 (27·94%)	1101 (24·83%)	403 (24·88%)	404 (24·94%)
**Only child**								
No	3820 (5·61%)	25,637 (37·66%)	3621 (20·73%)	3812 (21·83%)	911 (20·55%)	999 (22·53%)	366 (22·59%)	350 (21·60%)
Yes	5291 (7·77%)	33,323 (48·95%)	5111 (29·27%)	4920 (28·17%)	1306 (29·45%)	1218 (27·47%)	444 (27·41%)	460 (28·40%)
**Left behind experience**								
No	5537 (8·13%)	43,985 (64·62%)	5307* (30·39%)	6527 (37·37%)	1300 (29·32%)	1640 (36·99%)	454* (28·02%)	606 (37·41%)
Yes	3574 (5·25%)	14,975 (22·00%)	3425* (19·61%)	2205 (12·63%)	917 (20·68%)	577 (13·01%)	356* (21·98%)	204 (12·59%)
**Existing Diagnoses**								
Depression	266 (0·39%)	179 (0·26%)	259* (1·48%)	22 (0·13%)	129* (2·91%)	10 (0·23%)	66* (4·07%)	4 (0·25%)
GAD	343 (0·50%)	575 (0·84%)	330* (1·89%)	91 (0·52%)	129* (2·91%)	21 (0·47%)	64* (3·95%)	7 (0·43%)
OCD	465 (0·68%)	1022 (1·50%)	448* (2·57%)	157 (0·90%)	119* (2·68%)	39 (0·88%)	59* (3·64%)	13 (0·80%)
Schizophrenia	27 (0·04%)	36 (0·05%)	26* (0·15%)	5 (0·03%)	11 (0·25%)	1 (0·02%)	5 (0·31%)	0 (0·00%)
Bipolar disorder	61 (0·09%)	56 (0·08%)	57* (0·33%)	11 (0·06%)	26* (0·59%)	4 (0·09%)	14* (0·86%)	0 (0·00%)
Other mental disorders	122 (0·18%)	200 (0·29%)	118* (0·68%)	21 (0·12%)	44* (0·99%)	11 (0·25%)	17* (1·05%)	0 (0·00%)

*Note* Data are mean (SD) or n (%). Cases are participants endorsed corresponding suicidal thoughts or behaviors while controls are not. The cases in the total sample are individuals reported lifetime suicidality. Definitions and measuring instruments of all variables are shown in the Appendix (p.3–7). For diagnoses, GAD = generalized anxiety disorder, OCD = obsessive compulsive disorder. * Denotes significant group (*p* < 0·05) differences between the cases and controls after applying the Bonferroni correction

**Fig. 1:**
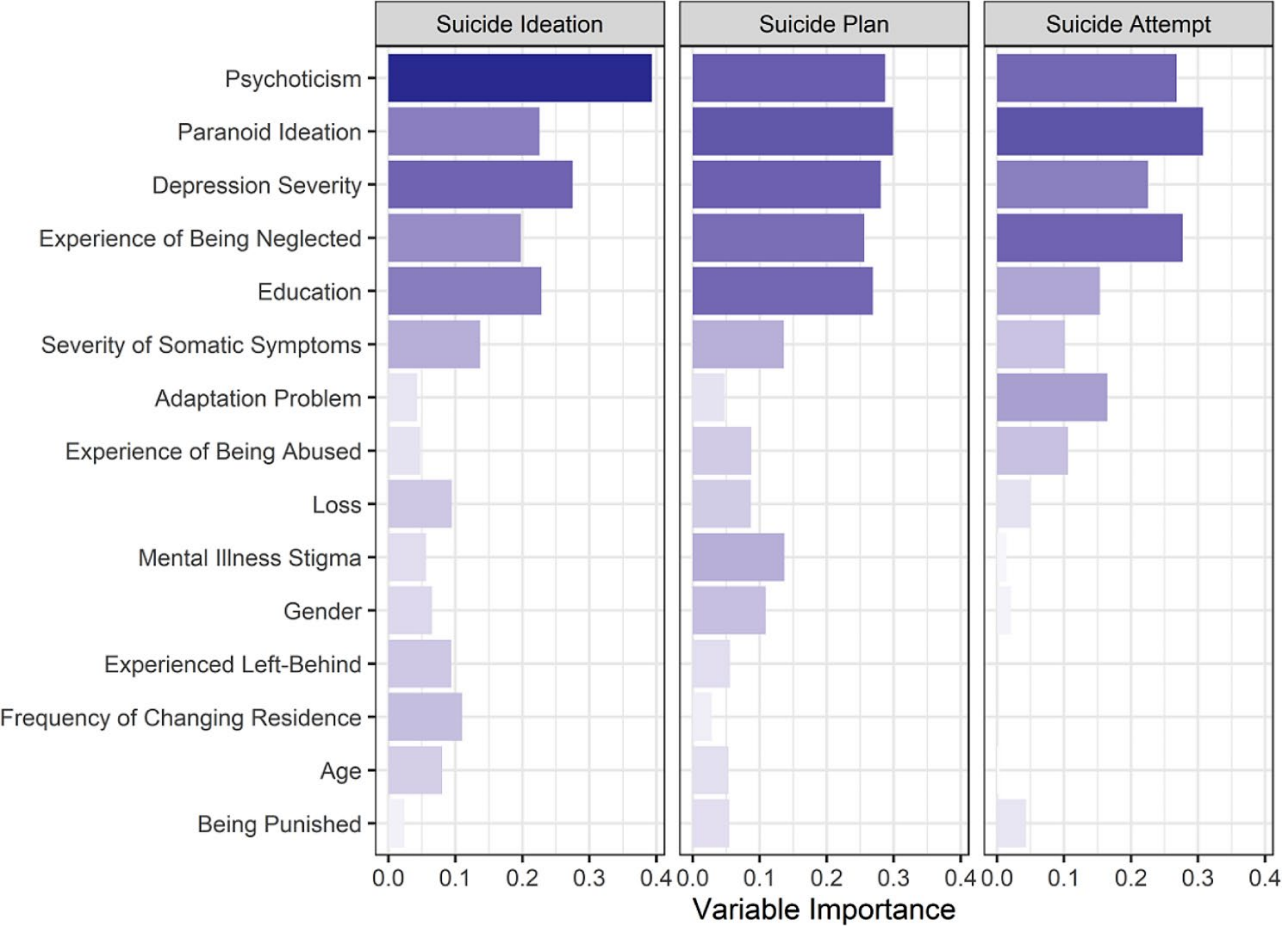
Variable importance based on mean absolute SHAP values Bar graphs showing the variable importance indicated by mean absolute SHAP values. The darker the color and the longer the length of the bar, the more important the variable is in predicting the corresponding suicidal behavior. From top to bottom, the variables are arranged according to averaged mean absolute SHAP values for the three suicidality

The balanced datasets were constructed to train XGBoost models, resulting in three datasets of 17,464, 4,434, and 1,620 samples for SI, SP, and SA, respectively. Based on the validation sets, the final models achieved good discrimination (AUC > 0·80, see Appendix Table A2), ensuring reliable associations between features and responses, and allowing the calculation of SHAP values for each feature. The list of variables used for training and their importance can be found in Appendix Table A3. The top 15 features with the highest averaged mean absolute SHAP values for the three suicide variables are showed in Fig. 1. Among the top three suicide-associated factors, both psychoticism (mean |SHAP| = 0·39) and depression severity (mean |SHAP| = 0·28) appeared to be the most significant predictors of SI. In contrast, paranoid ideation is the most significant factor for predicting SP (mean |SHAP| = 0·30) and SA (mean |SHAP| = 0·31). Additionally, childhood neglect has a differential effect on SA (mean |SHAP| = 0·28).

**Fig. 2:**
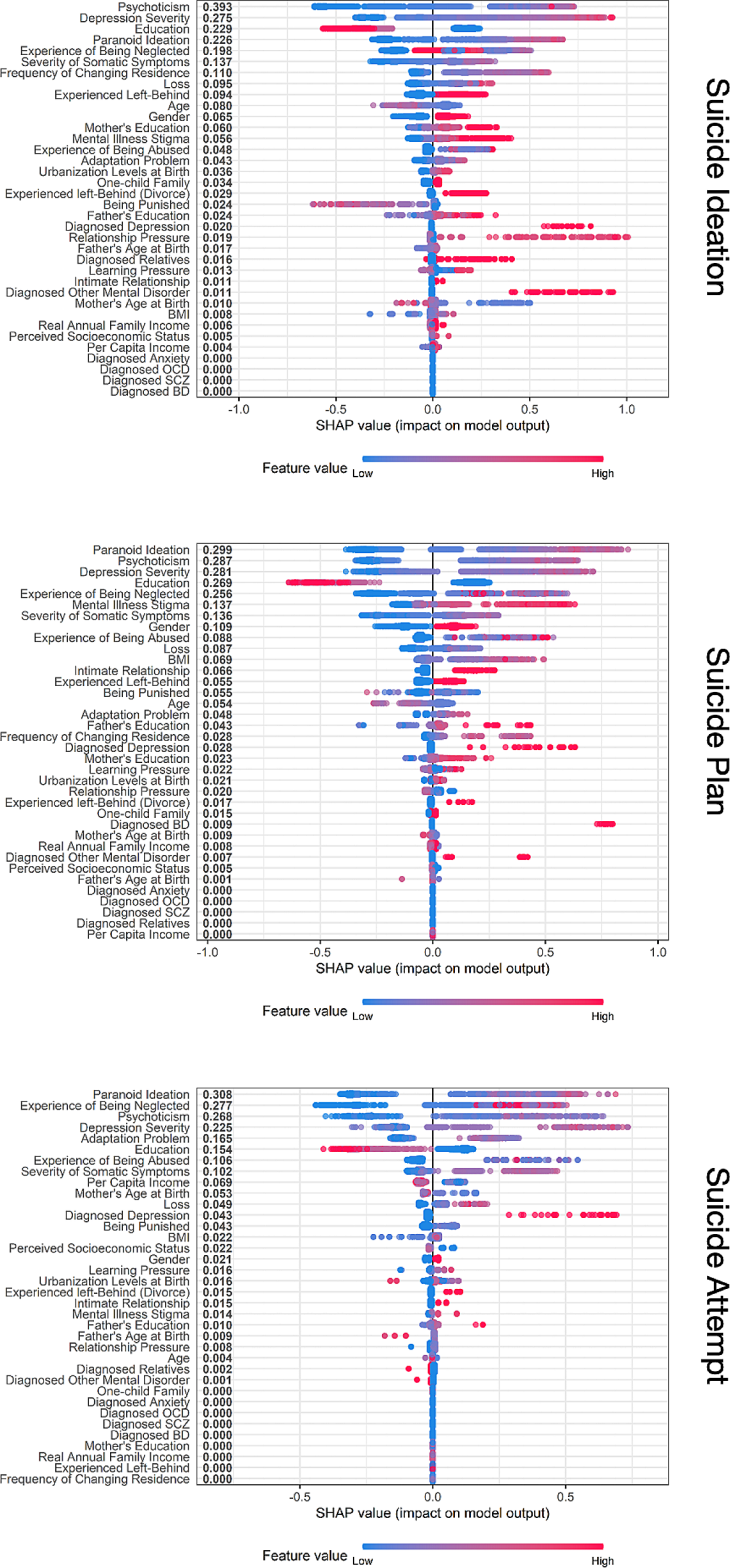
SHAP summary plot of suicide ideation, suicide plan and suicide attempt The figure ranks variable importance based on mean SHAP value magnitude which is juxtaposed to the variable names. For each variable, each dot corresponds to the SHAP value (log odds of suicide ideation/plan/attempt) of a participant and the color represents the value from low (blue) to high (red). A positive value increases the risk of suicidal ideation/plan/attempt

In the SHAP summary plot (Fig. 2), the associations between suicidal-related factors and suicidality are further illustrated. Generally, the features have similar trends in influencing suicidality across all three variables. For example, psychopathology factors are the strongest predictors, with psychoticism, depression severity, paranoid ideation ranking above other familial and life event factors. Additionally, several factors, such as being diagnosed with depression, experiencing relationship pressures, and having other mental disorders, revealed long right tails, suggesting they drastically increase the risk of SI for certain individuals. Interestingly, the only protective factor identified was education level, as higher education levels were associated with a lower chance of developing SI.

**Fig. 3:**
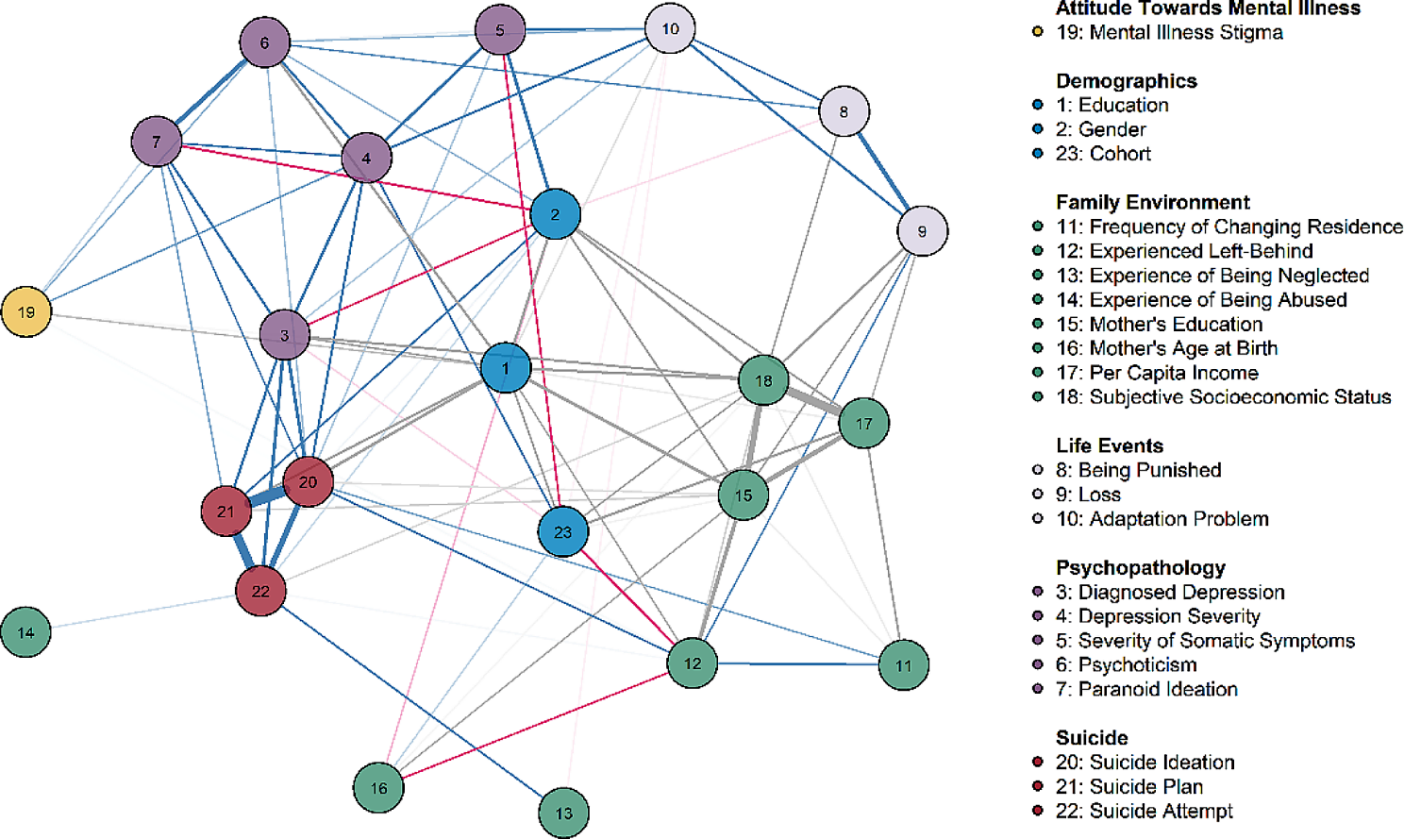
Graphic depiction of the suicidal behavioral network Visualization of the estimated mixed graphical model. The cluster (category) each node belongs to is specified in advance (see Appendix Table A3). Nodes are placed based on the Fruchterman-Reingold algorithm, and thus nodes that are less central are located at the periphery of the network. Thicker and darker edges represent stronger associations. Blue edges indicate positive associations and red edges indicate negative ones. Grey edges represent associations between categorical variables with more than two levels. These associations were characterized by more than one parameter and thus the sign (positive or negative) could not be defined

To examine the effect of suicide-associated factors holistically, we estimated a mixed graphical model (MGM) using suicidal variables and the top 15 factors with the highest mean absolute SHAP values for each suicide behavior. To simplify the graph, we included only gender and education as demographic variables and added a cohort variable to explore the temporal effects on other factors, resulting in a total of 23 variables. The MGM based on the total sample is depicted in Fig. 3. A first glance at the network suggested that most factors and suicidality were closely connected. With a few exceptions, experiences of abuse and neglect were uniquely associated with SA. A preliminary visual examination of the node locations further revealed a hierarchical structure of factors. Factors under the same category formed three interrelated clusters: family environment, psychopathology, and adverse life events. Furthermore, these three clusters, along with the attitude factor and demographics, revealed a hierarchical pattern of the trajectories of suicidality. The family environment cluster served as distal factors but could directly increase the risk of suicide behavior without resorting to other factors. Another cluster, life events that also include distal factors, may contribute to suicidality indirectly via more proximal psychopathology factors. Despite its marginal status, mental illness stigma affected a wide range of psychopathology factors.

**Fig. 4:**
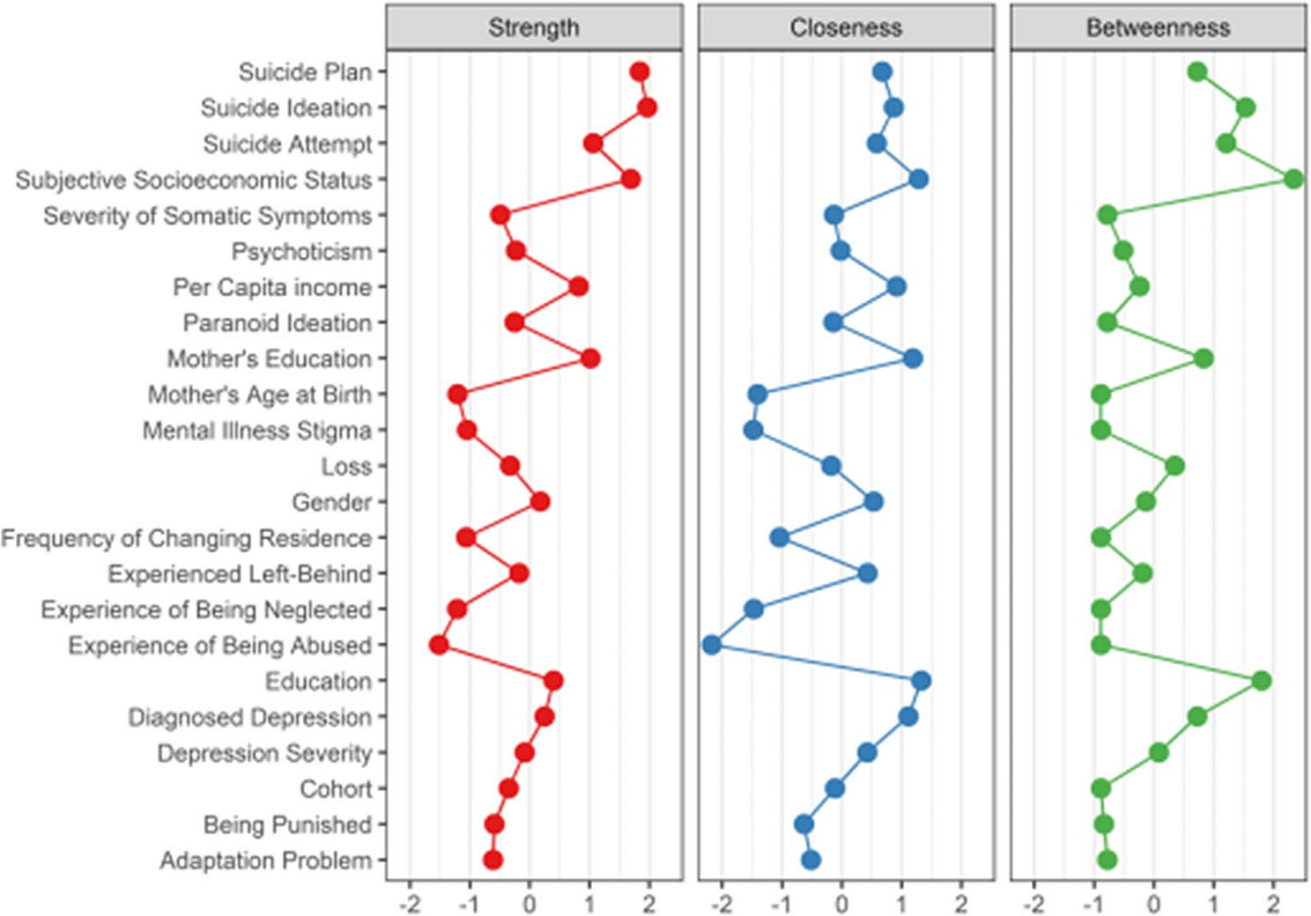
Strength, closeness, and betweenness centrality indices of suicidal behavior and factors The three most popular centrality indices were plotted for each node. All centrality indices were converted to z-scores. Nodes with a higher centrality are more important in the network

The relative importance of factors was examined using centrality indices. The high strength centrality of the three suicide variables (Fig. 4), particularly SA, suggested that they were largely related to other factors. The highest betweenness score of SA (z = 1·53) among the three highlighted its role as an intermediary between suicide-associated factors and suicidal behavior. As indicated by the strength centrality, subjective socioeconomic status (z = 1·68) appeared to be the most important node on the network for suicide-associated factors. In terms of closeness and betweenness centrality, education and subjective socioeconomic status emerged as the most influential factors. Education displayed a z-score of 1.33 for closeness and 1.80 for betweenness, while subjective socioeconomic status showed a z-score of 1.28 for closeness and 2.33 for betweenness. This indicates that these factors have a broad and rapid psychological impact, easily reaching other nodes within the network.

## Discussion

The current retrospective study, encompassing the largest cohort of young individuals aged 15–35 to date, endeavors to elucidate the intricate effects of suicide-associated factors. Leveraging interpretable machine learning and network analyses, our approach adopts both local and global perspectives based on multifactorial data. Our findings show that around 13% of young people in this age group experience suicidality. Based on SHAP values, psychopathology factors emerge as the strongest predictors of suicidality in this age group, with psychoticism and depression severity being primary predictors of SI, while paranoid ideation predominates in SP and SA. Neglect also emerges as a significant predictor for SA. Referring to MGM, there is a hierarchical structure among the suicide-associated factors. Specifically, family environments and life events serve as distal factors, preceding more proximal psychopathology factors. Notably, family environment factors retain direct effects on suicidality. Subjective socioeconomic status and education emerge as pivotal factors within the network, exerting broad influences on other factors through playing associative, mediating, and communicating roles. Overall, the study highlights the need for ongoing efforts to prevent suicidality in young people, focusing on addressing psychopathology, family environments, and adverse life events.

The study’s results have implications for public health initiatives targeting suicide prevention in young people. In Chinese young people, the prevalence rates of SI, SP, and SA were 12·8%, 3·3%, and 1·2%, respectively, with lower rates of SP and SA compared to previous studies [3, 5], which may be due to improvements in living conditions and decreased availability of lethal pesticides in rural areas [6, 23]. Additionally, suicidality in this age group is dominated by SI, while SP and SA are rare. This pattern is generally consistent with previous studies in China [3, 9], as suicide ideation is generated without any actual methods and materials.

One noteworthy finding highlighted in the SHAP summary plot warrants further discussion. It appears that across SI, SP, and SA, there is an increase in the risk of suicide associated with parents’ educational level. This seemingly counterintuitive association aligns with findings from a study conducted among Chinese college students [24]. parents with higher educational levels, often employed in professions such as teaching, healthcare, or civil service, tend to adopt authoritative parenting styles characterized by strict control. This authoritarian approach may diminish children’s sense of life value and psychological well-being, potentially contributing to suicidal tendencies.

Employing network analysis, the current study offers novel insights into suicide prediction and intervention by revealing the clustering and hierarchical relationships among suicide-related factors in an associative network. An empirical study supported the notion of a complex interrelationship among suicide risk factors, demonstrating that incorporating complexity into prediction models can substantially enhance the predictive accuracy of suicide risk factors [15]. Another recent study employed machine learning techniques confirmed the efficacy of a hierarchical multi-factor prediction method for suicide risk (Facebook texts → personality traits → psychosocial risks → psychiatric disorders → suicide) compared to a single-factor prediction method (Facebook texts → suicide), supporting the notion of a hierarchical relationship among suicide factors [24]. The results of present network analysis align with and consolidate these findings. The MGM reveals an interconnected network of suicide-related factors clustered into distinct groups, mirroring established empirical categories. For example, diagnosed depression, psychotic symptoms, and other psychopathology factors are tightly connected to each other. Building upon this, a two-level hierarchical structure emerges, comprising proximal and distal factors. Different distal factors interact with various proximal factors, leading to diverse pathways that impact suicidality. For example, distal factor 8 amplifies its influence on SI by affecting proximal factor 6 (being punished → psychoticism → SI → SP/SA). Simultaneously, it will also heightens the effect on proximal factor 3 by influencing distal factor 18, ultimately contributing to suicidality (being punished → subjective socioeconomic status → diagnosed depression → SI/SP/SA). This finding aligns with the well-established stress-diathesis model [11], where distal factors, especially those stemming from the family environment, heighten vulnerability to suicidality (the diathesis), while psychopathological factors act as triggers (the stressor).

In the MGM, psychopathology factors, as a proximal cluster, are the most powerful predictors of suicidal thoughts and behaviors. This aligns with the results of a recent meta-analysis that synthesized research spanning five decades, demonstrating a robust association between psychopathology and suicide across various age groups [12]. Our findings further reinforce this relationship by emphasizing the central role of psychopathology factors in predicting suicide within the 17–24 age group, extending previous findings in a smaller sample of U.S. participants aged 9-10 [13].

The MGM highlighted two specific risk factors for SA, classified as distal family factors within established empirical knowledge: being neglected and being abused. These factors bypassed proximal factors to directly influence SA. Previous studies have highlighted the strong correlation between these two factors and SA [25], with impulsivity emerging as a crucial contributing factor [26]. Since impulsivity can lead to immediate SA [27], the combination of these findings implies that suicide-related factors that are highly correlated with impulsivity may form an unique proximal cluster for SA if broader factors are considered.

The current study identified a diverse array of distal factors within the associative network, encompassing family dynamics, life event, demographics, and stigma. These findings provide further support for the implementation of broad school- and community-based intervention strategy. Family factors are more fundamental as distal factors to increase suicidal risk indirectly through adverse life events and psychopathology [13]. Moreover, familial influences extend beyond well-documented psychological factors such as family conflict and maltreatment [28], encompassing objective sociodemographic indicators of family environments and composition, may exhibit lower incremental validity in predicting suicidal behaviors, as indicated by their ranking in SHAP plots, they offer insights into the incubation of suicidal behaviors over extended periods and the childhood stressors associated with them. For example, as shown in the lower right corner of the MGM, these distal risk factors could be frequent changes in residence and lower incomes that suggest unstable and harsh childhood environments.

Based on centrality indices, subjective socioeconomic status (SES) is another notable distal factor, exhibiting the highest strength centrality within the network due to its extensive associations with both psychopathological and life event factors. Prior studies have found that subjective SES was associated not only with various future health outcomes but also different inheritance patterns within family history [29, 30]. Children who perceive themselves occupying lower ranks of a social hierarchy are susceptible to impairments of the brain’s stress regulatory systems, resulting in dysfunction in coping with long-term physiological and behavioral stress throughout life, particularly during adolescence [31]. The current results extend these findings by demonstrating that long-term consequences of low SES also include fatal suicidal behavior. The potential underlying mechanism is implied in its gatekeeper position (high betweenness) in the network. As a psychological indicator of objective family factors, subjective SES integrates influences from family environments and childhood maltreatment, consequently contributing to adverse life events and psychopathology, which serve as more proximate risk factors.

Similar to subjective SES, education emerges as another notable distal factor with high closeness and betweenness indices. Based on its location and its high betweenness in the network, education may influence suicide through the same mechanism as subjective SES. Notably, education may provide additional insights into family environments and SES beyond those captured by subjective social hierarchy, particularly as our assessment of subjective SES focuses primarily on income level. The recent decline in suicide rates in China has been attributed in part to increased educational opportunities spurred by urbanization, which affords people greater chances to overcome poverty and familial discord [6]. These mechanisms underscore that suicidal behavior transcends individual psychological crises, representing a broader societal issue entwined with economic factors such as income inequality, social comparisons, and childhood environments.

This study presents several strengths and limitations. The utilization of a large and homogenous sample, coupled with a comprehensive assessment range through the Online Health Survey (OHS), offers unique advantages in accurately detecting and quantifying suicide-related factors. Network analysis further enables us to discern clustered and hierarchical relationships among these factors, providing a holistic understanding from both local and global perspectives. However, potential recall bias in measures of childhood experiences and family SES may impact the statistical accuracy of our findings. Additionally, the overrepresentation of a single on-campus population could potentially limit the generalizability of our findings, hindering our ability to extrapolate the influence and signifcancce of risk factors to broader populations or different regions.

## Conclusions

In conclusion, our study has significant implications for research and clinical practice. To improve future research, we suggest employing multi-factorial study designs with rigorous ethical discussions and advanced statistical techniques that examine the contribution of each suicide risk factor. For clinical practice, we recommend adopting integrated multi-level efforts within a social-ecological model that considers risk factors at different levels. Our inferred hierarchical structure, while preliminary, highlights the importance of addressing psychopathology as the most critical factor and managing adverse family environment and negative life events in the long-term. However, interventions targeting only psychopathology may be insufficient in mitigating familial factors’ impact on suicidality, given their unique influence rooted in social inequalities and neural/physiological functions in coping with stress.

### Electronic supplementary material

Below is the link to the electronic supplementary material.

Supplementary Material 1

## Data Availability

The datasets generated during and/or analyzed during the current study are not publicly available due to ethical restrictions regarding data protection issues and the study-specific consent text and procedure, but anonymized data are available from the corresponding author upon reasonable request.

## Abbreviations

SI: Suicide ideation
SP: Suicide plan
SA: Suicide attempt
AUC: Area under the receiver operating characteristic curve
OHS: Online Health Survey system
CIDI: Chinese version of the World Mental Health Composite International Diagnostic Interview
SCL-90-R: Symptoms checklist-90-revised
PHQ-15: Patient health questionnaire-15
PHQ-9: Patient health questionnaire-9
ASLEC: Adolescent self-rating life events checklist
SHAP: Shapley additive explanation
MGM: Mixed graphical model
SD: Standard deviation
CI: Confidence intervals
SES: Subjective socioeconomic status
