# Supplementary material for: Risk factors and their association network for young adults’ suicidality: a cross-sectional study
Source: BMC Public Health. 2024 May 22;24:1378. doi: 10.1186/s12889-024-18860-9 (PMC11112863; doi:10.1186/s12889-024-18860-9)
Supplement: Supplementary file 1 — Supplementary Material 1 [file 12889_2024_18860_MOESM1_ESM.docx]

**Risk Factors and Their Association Network for Young Adults' Suicidality: A Cross-Sectional Study**

**Appendix**

**Methods**

1. **The Online Health Survey (OHS) study and sample selection**

**Figure A1. Flowchart of data analysis and interpretation**

1. **Definition of variables used in the current study**

**Table A1. Definition of variables used in the current study**

**Results**

**4. Detecting risk factors of suicide behaviors by XGBoost**

**Table A2. Accuracy of the final models of XGBoost**

**5. Examining and Interpreting the results of suicide-associated factors**

**Table A3. Feature categories and feature importance**

**Online Methods**

**1. The Online Health Survey (OHS) study and sample selection**

The Online Health Survey (OHS) system of Sichuan University is a mixed-method observational survey of all college students in Sichuan University, which aims to track the physical and mental health status of university students. Ethics oversight was provided by the Ethics Committee of West China Hospital, Sichuan University.

Sichuan University is a comprehensive university with students from all over the country. All enrolled freshmen from 2016 to 2020 were invited to participate in the OHS study. After accepting the invitation, participants would log on to the OHS website to engaged in the informed consent process, and subsequently begin to fill out the survey. During the filling process, participants could opt-out at any time. After excluding abnormal samples (i.e., uncompleted surveys, completed surveys that took significantly less time than average, completed surveys with obviously abnormal information), 68071 (99.4%) of the 68472 samples were finally included.

All questionnaires and scales used in the OHS survey are self-reported, selected from validated and published measurement tools by a professional team of clinicians, psychiatrists, psychologists. The survey broadly included measures of factors related to the subjects' physical and health status, including the Chinese version of the World Mental Health Composite International Diagnostic Interview (CIDI),^1^ Symptoms checklist-90-revised (SCL-90-R),^2^ the Patient Health Questionnaire-15 (PHQ-15),^3^ the Patient Health Questionnaire-9 (PHQ-9),^4^ and the adolescent self-rating life events checklist (ASLEC).^5^ Sociodemographic factors for recruiting samples included age, gender, socioeconomic status, and urbanicity. Ethics oversight was provided by the Ethics Committee of West China Hospital, Sichuan University.

**Figure A1. Flowchart of data analysis and interpretation**

**Model training and testing**

Select optimal hyperparameters of XGBoost based on grid search with 5-fold cross-validation. Performance is evaluated based on validation sets.

**Identify important factors**

Mean absolute SHAP values are calculated for each variable based on the final models. Variables with higher mean absolute SHAP values are more important in predicting suicidal behavior.

**Local explanations**

1. Rank feature importance to identify proximate risk factors of each suicidal behavior.

2. Explain the effects of individual factors based on the SHAP summary plot.

**Global explanations**

1. A mixed graphical model was estimated by the 15 most important factors for each suicide behavior.

2. Combined with local explanations, explain the relationships between proximal and distal risk factors in order to trace the trajectories of suicidal behavior.

**Preprocessing**

1. Remove anomaly cases

2. Conduct descriptive analysis

3. Construct balanced data for each suicidal behavior

Data

Modeling

Identification

Explaining

**2. Definition of variables used in the current study**

| **Table A1. Definition of variables used in the current study** | | |
| --- | --- | --- |
| **Variable** | **OHS Survey** | **Description** |
| **Suicidal Thoughts and Behaviors** | | |
| Suicide ideation | The suicidal ideation and behaviors module from the computerized CIDI | Categorical data, the presence of suicidal ideation during lifetime, in the cleansed dataset coded as:  0=no  1=yes |
| Suicide plan | The suicidal ideation and behaviors module from the computerized CIDI | Categorical data, the presence of suicidal plan during lifetime, in the cleansed dataset coded as:  0=no  1=yes |
| Suicide attempt | The suicidal ideation and behaviors module from the computerized CIDI | Categorical data, the presence of suicidal attempt during lifetime, in the cleansed dataset coded as:  0=no  1=yes |
| **Demographics** | | |
| Age | OHS Demographics | Gaussian data, chronological age |
| Gender | OHS Demographics | Categorical data, responses were coded as:  1=Male  2=Female |
| Education | OHS Demographics | Categorical data, responses were coded as:  1=Undergraduate  2=Postgraduate  3=Doctorate |
| Intimate relationship | OHS Demographics | Categorical data, in the cleansed dataset coded as:  1=not yet  2=yes |
| Only child family | OHS Demographics | Categorical data, responses were coded as:  1=yes  2=no |
| Body mass index (BMI) | OHS Demographics | Gaussian data, Body Mass Index:  The body mass divided by the square of the body height (BMI=kg/m^2^). |
| **Family Environment** | | |
| Father's education | OHS Demographics | Categorical data, in the cleansed dataset coded as:  1=Illiteracy  2=Elementary school  3=Junior high school  4=High school  5=College  6=Undergraduate  7=Postgraduate  8=Doctorate |
| Father's age at birth | OHS Demographics | Gaussian data, chronological age |
| Mother's education | OHS Demographics | Categorical data, in the cleansed dataset coded as:  1=Illiteracy  2=Elementary school  3=Junior high school  4=High school  5=College  6=Undergraduate  7=Postgraduate  8=Doctorate |
| Mother's age at birth | OHS Demographics | Gaussian data, chronological age |
| Subjective socioeconomic status | OHS Demographics | Categorical data, responses were coded as: 1=low income  2=lower middle income  3=middle income  4=upper middle income  5=high income |
| Real annual family income | OHS Demographics | Categorical data, responses were coded as:  1=less than ¥5,000  2=between ¥5,000 and ¥10,000  3=between ¥10,000 and ¥30,000  4=between ¥30,000 and ¥50,000  5=between ¥50,000 and ¥100,000  6=more than ¥100,000 |
| Per capita income | OHS Demographics | Categorical data, responses were coded as:  1=less than ¥3,000  2=between ¥3,000 and ¥5,000  3=between ¥5,000 and ¥10,000  4=between ¥10,000 and ¥20,000  5=between ¥20,000 and ¥30,000  6=more than ¥30,000 |
| Urbanization levels at birth | OHS Urbanization Survey | Categorical data, responses were coded as:  1=village  2=town  3=county-level city  4=prefecture-level city  5=direct-administered municipality |
| Frequency of changing residence | OHS Urbanization Survey | Gaussian data, the frequency of changing residence |
| Experienced left-behind | OHS left-behind experiences questionnaire | Categorical data, whether the participant has an experience in which one/both parents are not around for at least 6 months, responses were coded as:  1=yes  2=no |
| Experience left behind (divorce) | OHS left-behind experiences questionnaire | Categorical data, whether the participant has an experience in which one/both parents are not around for at least 6 months caused by parents’ divorce, in the cleansed dataset coded as:  0=no  1=yes |
| Experiences of being neglected | The Experiences of Being Neglected extracted from the computerized CIDI | Gaussian data |
| Experiences of being abused | The Experiences of Being Abused extracted from the computerized CIDI | Gaussian data |
| **Adverse Life Events** | | |
| Experiences of being punished | Based on the factor of Being Punished extracted from ASLEC | Gaussian data, included ASLEC item:  18, 19, 20, 21, 23, 24 and 26 |
| Experiences of loss (losing) | Based on the factor of Loss extracted from ASLEC | Gaussian data, included ASLEC item:  11, 12, 13, 14, 16 and 17 |
| Interpersonal pressure | Based on the factor of Interpersonal Pressure extracted from ASLEC | Gaussian data, included ASLEC item:  1, 2, 4 and 15 |
| Learning pressure | Based on the factor of Learning Pressure extracted from ASLEC | Gaussian data, included ASLEC item:  3, 9, 22 and 25 |
| Adaption problem | Based on the factor of Adaption Problem extracted from ASLEC | Gaussian data, included ASLEC item:  18, 19, 20, 21, 23, 24 and 26 |
| **Psychopathology** | | |
| Diagnosed depression | OHS Medical History Questionnaire | Categorical data, responses were coded as:  1=yes  0=no records |
| Diagnosed generalized anxiety disorder | OHS Medical History Questionnaire | Categorical data, responses were coded as:  1=yes  0=no records |
| Diagnosed obsessive compulsive disorder | OHS Medical History Questionnaire | Categorical data, responses were coded as:  1=yes  0=no records |
| Diagnosed schizophrenia | OHS Medical History Questionnaire | Categorical data, responses were coded as:  1=yes  0=no records |
| Diagnosed bipolar disorder | OHS Medical History Questionnaire | Categorical data, responses were coded as:  1=yes  0=no records |
| Diagnosed other mental disorders | OHS Medical History Questionnaire | Categorical data, responses were coded as:  1=yes  0=no records |
| Family history of mental illness (diagnosed relatives) | OHS Medical History Questionnaire | Categorical data, and for each diagnosed mental disorder responses were coded as:  1=yes  0=no records |
| Depression severity | Based on the PHQ-9 score | Gaussian data |
| Severity of somatic symptoms | Based on the PHQ-15 score | Gaussian data |
| Psychoticism | Based on the factor of Psychoticism extracted from SCL-90 | Gaussian data, included SCL-90 item:  7, 16, 35, 62, 77, 84, 85, 87, 88, and 90 |
| Paranoid ideation | Based on the factor of Paranoid Ideation extracted from SCL-90 | Gaussian data, included SCL-90 item:  8, 18, 43, 68, 76, and 83 |
| **Stigma** | | |
| Mental illness stigma | The score extracted from the OHS Questionnaire on Attitudes towards Psychological Problems and Mental Illness | Gaussian data |

**3. Local Explanations and Global Understanding of Suicide-Associated Factors**

We used Shapley additive explanation (SHAP) values and mixed graphical model (MGM) to explore a a more comprehensive understanding of the relationship between suicide-associated factors and suicidality.

SHAP values were developed to interpret black-box models like XGBoost and it provide several advantages in revealing complex relationships between factors and suicide. First, SHAP values of all features (factors) for each observation sum up to the model output (e.g., log odds of suicidal behavior), making the impact of individual factors interpretable while still capturing global patterns of the complex model. Second, SHAP values satisfy a consistency property that makes it legitimate to compare feature importance across models. In this study, mean absolute SHAP values for each feature in each model were calculated to compare the relative importance of various factors in predicting suicide ideation, plan, and attempt. Then, the individual SHAP values of each feature were plotted in a SHAP summary plot for each observation to show the distribution of feature contributions to the model out. The summary plot provided a clear relationship between these factors and suicidal behavior.

MGM is a regularized network estimation method and estimate parsimonious and interpretable relationships between variables.^6^ Each node in the MGM represents a factor, and each edge between two nodes represents a non-zero association. To reduce spurious associations, the MGM uses graphical LASSO regularization and EBIC model selection. The top 15 factors with the highest mean absolute SHAP values for each suicidal behavior were used to estimate the MGM. Three centrality indices (strength centrality, closeness centrality, and betweenness centrality) were calculated for each node to measure its importance in the network. The strength centrality describes the strength of association between the node and other nodes as a whole. The closeness centrality measures the ability of one node to spread information to other nodes quickly. The betweenness centrality measures how likely a node is to be a boundary spanner between other nodes. The MGM provided insight into the hierarchical distribution and interaction of suicide-associated factors, beyond individual factor contributions.

**Online Results**

**4. Detecting risk factors of suicide behaviors by XGBoost**

| **Table A2. Accuracy of the final models of XGBoost** | | | | |
| --- | --- | --- | --- | --- |
|  | AUC | Sensitivity | Specificity | F1 score |
| Suicide Ideation | 0·81 | 0·76 | 0·71 | 0·74 |
| Suicide Plan | 0·82 | 0·74 | 0·74 | 0·74 |
| Suicide Attempt | 0·83 | 0·73 | 0·80 | 0·76 |

Note: Sample sizes of validation sets for suicide ideation, plan, and attempt are 3492, 886, and 324, respectively. Due to random initialization (the random undersampling process), the results may vary slightly.

**5. Examining and Interpreting the results of suicide-associated factors**

| **Table A3. Feature categories and feature importance** | | | | | | |
| --- | --- | --- | --- | --- | --- | --- |
|  | **Number of features** | **Number of features with average importance in the top 15** | **Number of features with importance in the top 15 (ideation)** | **Number of features with importance in the top 15 (plan)** | **Number of features with importance in the top 15 (attempt)** | **Feature names** |
| Demographic | 6 | 3 | 3 | 5 | 2 | Age; gender; education; intimate relationship; only child; body mass index (BMI) |
| Family environment | 13 | 4 | 5 | 3 | 5 | Father’s education; father’ age at birth; mother’s education; mother’s age at birth; subjective socioeconomic status; real annual family income; per capita income; urbanization levels at birth; frequency of changing residence; experienced left-behind; experience left behind (divorce); experience of being neglected; experience of being abused |
| Adverse life event | 5 | 3 | 2 | 2 | 3 | Being punished; losing; Interpersonal pressure; learning pressure; adaptation problem |
| Psychopathology | 11 | 4 | 4 | 4 | 5 | Diagnosed with depression, generalized anxiety disorder, obsessive compulsive disorder, schizophrenia, bipolar disorder, other mental disorders; diagnosed relatives; depression severity; severity of somatic symptoms; psychoticism; paranoid ideation |
| Stigma | 1 | 1 | 1 | 1 | 0 | Mental illness stigma |

Note: Feature names are variables included in the current study. The number of variables in the top 15 features ranked by mean absolute SHAP values are shown for each suicidal behavior.

**References**

1. Sartorius N, Janca A. Psychiatric assessment instruments developed by the World Health Organization. *Soc Psychiatry Psychiatr Epidemiol*. 1996;31(2):55-69.

2. Derogatis LR. *Symptoms Checklist-90. Administration, Scoring, and Procedures Manual for the Revised Version*.; 1977.

3. Kroenke K, Spitzer RL, Williams JBW. The PHQ-15: Validity of a New Measure for Evaluating the Severity of Somatic Symptoms: *Psychosom Med*. 2002;64(2):258-266.

4. Kroenke K, Spitzer RL, Williams JBW. The PHQ-9: Validity of a brief depression severity measure. *J Gen Intern Med*. 2001;16(9):606-613.

5. Xin X, Yao S. Validity and reliability of the Adolescent Self-rating Life Events Checklist in middle school students. *Chin Ment Health J*. 2015;(5):355-360.

6. Epskamp S, Fried EI. A Tutorial on Regularized Partial Correlation Networks. *Psychol Methods*. 2018;23(4):617-634.
